# Old Question Revisited: Are High-Protein Diets Safe in Pregnancy?

**DOI:** 10.3390/nu13020440

**Published:** 2021-01-29

**Authors:** Thorhallur I. Halldorsson, Bryndis E. Birgisdottir, Anne L. Brantsæter, Helle Margrete Meltzer, Margaretha Haugen, Inga Thorsdottir, Anna S. Olafsdottir, Sjurdur F. Olsen

**Affiliations:** 1 Faculty of Food Science and Nutrition, School of Health Sciences, University of Iceland, 101 Reykjavik, Iceland; beb@hi.is (B.E.B.); ingathor@hi.is (I.T.); 2 Centre for Foetal Programming, Department of Epidemiology Research, Statens Serum Institut, 2300 Copenhagen, Denmark; SFO@ssi.dk; 3 Division of Infection Control and Environmental Health, Norwegian Institute of Public Health, 0213 Oslo, Norway; annelise.brantsaeter@fhi.no (A.L.B.); hellemargrete.meltzer@fhi.no (H.M.M.); almargar@online.no (M.H.); 4 Faculty of Health Promotion, Sport and Leisure Studies, School of Education, University of Iceland, 105 Reykjavik, Iceland; annaso@hi.is; 5 Department of Nutrition, Harvard TH Chan School of Public Health, Boston, MA 02115, USA

**Keywords:** DNBC, MoBa, diet, protein, pregnancy, complications, preterm birth, fetal growth, perinatal mortality

## Abstract

Background: A previous randomized dietary intervention in pregnant women from the 1970s, the Harlem Trial, reported retarded fetal growth and excesses of very early preterm births and neonatal deaths among those receiving high-protein supplementation. Due to ethical challenges, these findings have not been addressed in intervention settings. Exploring these findings in an observational setting requires large statistical power due to the low prevalence of these outcomes. The aim of this study was to investigate if the findings on high protein intake could be replicated in an observational setting by combining data from two large birth cohorts. Methods: Individual participant data on singleton pregnancies from the Danish National Birth Cohort (DNBC) (*n* = 60,141) and the Norwegian Mother, Father and Child Cohort Study (MoBa) (*n* = 66,302) were merged after a thorough harmonization process. Diet was recorded in mid-pregnancy and information on birth outcomes was extracted from national birth registries. Results: The prevalence of preterm delivery, low birth weight and fetal and neonatal deaths was 4.77%, 2.93%, 0.28% and 0.17%, respectively. Mean protein intake (standard deviation) was 89 g/day (23). Overall high protein intake (>100 g/day) was neither associated with low birth weight nor fetal or neonatal death. Mean birth weight was essentially unchanged at high protein intakes. A modest increased risk of preterm delivery [odds ratio (OR): 1.10 (95% confidence interval (CI): 1.01, 1.19)] was observed for high (>100 g/day) compared to moderate protein intake (80–90 g/day). This estimate was driven by late preterm deliveries (weeks 34 to <37) and greater risk was not observed at more extreme intakes. Very low (<60 g/day) compared to moderate protein intake was associated with higher risk of having low-birth weight infants [OR: 1.59 (95%CI: 1.25, 2.03)]. Conclusions: High protein intake was weakly associated with preterm delivery. Contrary to the results from the Harlem Trial, no indications of deleterious effects on fetal growth or perinatal mortality were observed.

## 1. Introduction

Forty years ago, a trial with high-protein supplementation in pregnancy, often referred to as the Harlem Trial, was undertaken in New York City [1]. Women were recruited from a poor urban African American population who had one or more risk factors for having a low-birth weight infant. The supplementation consisted of 40 g casein in liquid form corresponding to a daily intake of ~100 g protein (or ~20% of energy intake). The stated objectives of the trial were to “accelerate fetal growth and raise birth weight, and to influence later development”. However, as described in the initial report [1], no beneficial effects were detected. Instead, the mean birth weight of infants born prior to 37th week of gestation was significantly reduced [1] and the risk of small-for-gestational age (SGA) was increased [2]. Increased risks of delivery prior to 30 weeks and neonatal deaths were also reported, although only on the verge of statistical significance [1]. The investigators concluded that “Review of all available published and unpublished data makes it clear that high density protein supplementation during pregnancy consistently retards fetal growth” [1]. Their recommendation was also clear: “high protein supplementation in pregnancy must surely be avoided, at least until better information becomes available” [1]. 

Ever since their initial publication, the findings from the Harlem Trial have had considerable influence on public health recommendations. As an example, the current WHO recommendations for antenatal care do not recommend high-protein supplementation in undernourished populations [3]. The results from this trial have also weighted heavily in dietary recommendations for well-nourished populations [4] and in conclusions of systematic reviews [2,5,6,7]. In contrast, observational studies in well-nourished populations [8,9,10] only reported a modest reduction in fetal growth at high maternal protein intakes. Those studies did not, however, address adverse outcomes such as fetal growth retardation and mortality. The recent wave of low-carbohydrate, high-protein ketogenic diets for weight loss [11,12] makes the case for reassessing the findings of the Harlem Trial of public health relevance. Observational studies sufficiently powered for such analyses are, however, rare. 

Using prospective data on 126,443 singleton pregnancies from two large Nordic cohorts, we were able to identify more than 17,000 women who reported a protein intake in mid-pregnancy of at least 100 g/d. Our aim was to see if the associations with adverse pregnancy outcomes observed in the Harlem Trial could be replicated in this setting. 

## 2. Materials and Methods 

### 2.1. Population and Study Design

The Danish National Birth Cohort (DNBC: recruitment all over Denmark in 1996–2002, conducted at the Statens Serum Institut, Denmark) and the Norwegian Mother, Father and Child Cohort Study (MoBa: recruitment all over Norway in 1999–2008, conducted by the Norwegian Institute of Public Health) are prospective population-based pregnancy cohorts [13,14]. During the recruitment period, around 101,042 pregnancies or 35% of all pregnant women in Denmark were recruited into the DNBC. In MoBa, around 40.6% of all pregnant women were recruited and in total, the cohort includes 95,200 mothers, 75,200 fathers and their 114,500 children.

In the DNBC, women were recruited during their first antenatal visit to the general practitioner (gestational week ~6), whilst in MoBa, pregnant women were invited to participate by mail prior to the first ultrasound examination in gestational week ~18. In both cohorts, a comparison between participants and non-participants from the general pregnant population has been performed, finding no indication of selection bias for selected birth outcomes, including preterm delivery and low birth weight [15,16].

The MoBa dataset used for this study is based on version four of the quality-assured datafiles made available in 2008. Quality assurance here means that the electronically scanned version of the file was checked to reflect the exact answers given by respondents, including improbable answers, leaving it to the researchers to decide which answers to exclude. The MoBa data contained information on singleton pregnancies among participants answering a questionnaire on lifestyle and health in gestation week ~15 (Q1) and a food frequency questionnaire (FFQ) in week 22 (Q2), in use from 2002 throughout the recruitment period. The DNBC data were from a quality-assured datafile containing information on women who answered an FFQ in mid-pregnancy and participated in two telephone interviews on lifestyle and health in gestational weeks 12 and 30. 

Written informed consent was obtained from all participants in both cohorts. The establishment and data collection in MoBa were previously based on a license from the Norwegian Data Protection Agency and approval from The Regional Committee for Medical Research Ethics (S-95113/S-97045). They are now based on regulations related to the Norwegian Health Registry Act. The regional scientific ethics committee for the municipalities of Copenhagen and Frederiksberg approved all DNBC study protocols (27 August 2013: H-2-2013-108). All procedures were in accordance with the Declaration of Helsinki.

### 2.2. Inclusion Criteria

In the DNBC, dietary information was available for a total of 70,183 pregnancies. Of those, 1078 and 1590 pregnancies were excluded due to unrealistically low (≤4.5 MJ) or high (>20.0 MJ) energy intake and multiple pregnancies, respectively. During recruitment, women could enter the study more than once, and by restricting our analyses to first pregnancy enrollment, an additional 6666 pregnancies were excluded. Additional exclusion of women with pre-existing diabetes resulted in the final DNBC dataset of 60,141 women.

The MoBa database contained information on 77,750 singleton pregnancies with available dietary information. Using the same energy restriction as for the DNBC, an additional 1221 pregnancies were excluded. Further restriction to first pregnancy enrollment (*n* = 9224) and exclusion of women with pre-existing diabetes (*n* = 1003) resulted in the final dataset of 66,302 women. The combined number of women available for analyses from both cohorts was 126,443. See the flowchart in Supplemental Figure S1.

### 2.3. Dietary Variables

Considerations for merging the dietary variables of the participants from both cohorts have been described elsewhere [17]. In both cohorts, the FFQs were designed and validated specifically for pregnant women [18,19,20,21].

The FFQs gave portion sizes for units of fruit, bread (slices) and liquids (cups/glasses). However, to scale frequencies of intake into amounts (g/d), both cohorts used assumptions of standard Norwegian and Danish portion sizes for women, gathered from national dietary surveys. Based on these assumptions and by defining recipes for composite foods, the amount consumed for each food item in the FFQs was quantified in g/day. The amount of energy and nutrients from each food item was then estimated based on linkage to the National Food Composition Table. The total amount of energy (in MJ/day), protein (in g/day) and other nutrients was then estimated by aggregating the contribution from all food items.

Portion sizes used for protein-rich food were similar in the two FFQs and the same software for nutrient calculations was used. The timeframe for the two FFQs covered was, however, slightly different. In DNBC, the FFQ was administered in gestational week ~25 and covered food intake over the previous four weeks, while in MoBa, the FFQ was administered in week 22 (week 17 up to 2004), covering food intake from the beginning of pregnancy.

### 2.4. Outcome Variables

Results from the Harlem Trial [1] suggested that high-protein supplementation may result in an excess risk of very early preterm deliveries, growth retardation up to week 37 of pregnancy and neonatal deaths. To address these outcomes, in our primary analyses, we examined associations between maternal protein intake and (1) birth weight as a continuous outcome among all infants and preterm (<37 weeks) infants only; (2) low birth weight (<2500 g), (3) preterm delivery (<37 weeks) and (4) fetal death, defined as death occurring after filling out the FFQ and until or during delivery; and (5) neonatal death, defined as offspring death occurring during the first 28 days of life. In our secondary analyses, associations with late (weeks 34 to <37), early (32 to <34 weeks) and very early (<32 weeks) preterm deliveries were also explored.

Information on birth weight and fetal death was extracted from the national birth registries and information on neonatal death was obtained from national death registries in both countries. In MoBa, gestational age (in days) was primarily (98%) based on the ultrasound measure from weeks 17 to 18. When that information was missing, information on last menstrual period as reported by the women during pregnancy (2%) was used. In DNBC, gestational age (in days) was assessed from the last menstrual period (43%), based on maternal report at recruitment (week ~6) or in the first telephone interview (week 12). If this estimate was uncertain due to irregular or abnormal (>32 or <24 days) menstrual cycles, gestational age was based on information on expected date of delivery (55%) as reported by the women, which was most often based on ultrasound scanning. If this information was missing, gestational age as assessed at delivery by the midwife was used (2%).

### 2.5. Covariate Information

The following set of covariates were selected a priori for adjusted models: cohort (0/1), maternal age (<20, 20–24, 25–29, 30–34, 35–39 and ≥40 years, no missing), parity (1, 2, 3+, 2.3% missing), energy intake (in quintiles, no missing), pre-pregnancy BMI (<18.5, 18.5–24.9, 25–29.9 and >30 kg/m^2^, 6.2% missing), maternal smoking during pregnancy (no, occasionally and daily smoking, 3.0% missing), maternal education (less than basic studies, high school, college or higher education, 15.1% missing), maternal height (<160, 160–165, 166–175 and >175 cm, 4.8% missing), maternal cohabitant status (single, non-single, 4.9% missing) and fetal sex (binary variable, 0.2% missing). Variables with missing values over 3% were assigned to a missing category and adjustments were made using dummy variables. However, if missing values were <3%, they were imputed using the mode from the variable’s distribution. To account for potential confounding for energy intake, maternal protein intake was adjusted for energy using the residual method [22]. That is, protein intake of participants was regressed on their total energy intake. The residuals were then scaled using a constant corresponding to the predicted protein intake for the mean energy intake. These scaled residuals reflect individual variation in protein as predicted by energy intake. This approach is comparable to using nutrient densities but is more effective in accounting for potential confounding by energy.

### 2.6. Statistical Analysis 

Continuous normally distributed variables were described using the mean and standard deviation (SD), while dichotomous variables were described using percentages. Associations with dichotomous and continuous outcomes were examined using multivariate logistic and linear regression analyses, respectively.

Maternal protein intake was divided into categories of <60, 60–70, >70–80, >80–90, >90–100 and >100 g/day. The most prevalent (34% of subjects) category of >80–90 g/day protein was used as the reference. The highest protein category reflects similar intakes as reported in the high-protein supplementation group in the Harlem Trial [1]. As a test for overall effect, we used a chi-square test (type III) for dichotomous and F test (type III) for continuous variables, testing the null hypothesis that the response across groups was equal.

In our secondary analyses, we explored associations at more extreme protein intakes of ≥105, ≥110, >115 and >120 g/day using the same reference as in the primary analyses. As the protein supplementation in the Harlem Trial was based on casein, we also explored associations between dairy protein (from milk, yoghurt, cheese and butter) with our primary outcomes separately. Finally, the stability of our preterm delivery estimates was examined by repeating our analyses using the gestational age estimate based on the date for last menstrual period that was partly available for both cohorts.

## 3. Results

The prevalence of preterm delivery, low birth weight and fetal and neonatal deaths was 4.77%, 2.93%, 0.28% and 0.17%, respectively, with similar prevalence being observed in both cohorts (Table 1). Mean birth weight (SD) was 3591 g (566 g). Maternal characteristics were generally similar, except for maternal smoking, which differed substantially between the two cohorts. In both cohorts, energy (mean 10.0 MJ) and protein intakes (mean 89 g/day) were similar. On average, more than two thirds (~60–70 g/day) of dietary protein came from animal sources, with the intake of proteins from dairy being relatively high (~30 g/day). After adjusting for energy [22], the overall protein distribution used in our analyses was 68, 79, 87, 103 and 109 g/day for the 5th, 25th, 50th, 75th and 95th percentiles, respectively.

Offspring of mothers with low protein intake (<60 g/day) had around 130 g lower birth weight compared to offspring whose mothers had modest (80–90 g/day) intake (Table 2). At higher maternal intakes (up to >100 g/day), no meaningful differences in birth weight were observed compared to mothers with modest (80–90 g/day) intakes. A similar pattern was observed when examining preterm (<37 weeks) infants only. 

In unadjusted analyses, the association between maternal protein intake and preterm delivery (Table 3) was U-shaped with an OR of 1.31 (95%CI: 1.04, 1.64) for preterm delivery in those with low intake (<60 g/day) and an increased OR of 1.11 (95%CI: 1.02, 1.21) for those with high (>100 g) compared to moderate (80–90 g/day) intake. After adjustment for covariates, the effect estimate for low (<60 g/day) intake was attenuated and non-significant but a similar and significant increased risk of 1.10 (95%CI: 1.01, 1.19) was still observed for high protein (>100 g/day) compared to moderate protein (80–90 g/day) intake. High (>100 g/day) protein intake was also not associated with low birth weight, while increased risk was observed at low protein intake (<60 g/day). Overall, the effect estimates for preterm delivery and low birth weight were similar in both cohorts (Supplemental Table S1). When stratifying by pre-pregnancy (<25 and ≥25 kg/m^2^), similar associations between protein intake and preterm delivery and low birth weight were also observed in both strata (Supplimental Table S2).

As secondary analyses, we also examined the association with birth weight and preterm delivery at more extreme protein intakes. Compared to moderate protein intake (80–90 g), no indication of reduced birth weight among all infants or preterm infants only was observed at intakes as high as >120 g (Table 4).

The effect estimates for preterm delivery at high protein intake (see Table 3) were also stable around 1.10 at intakes up to >120 g/day (Table 5) and a non-significant increase in risk of low birth weight was observed at these high intakes.

Maternal protein intake was not associated with fetal death or neonatal death (Table 6, p for effect >0.20 in all cases). A non-significant decreased risk of fetal death was observed at high (>100 g) vs. modest (80–90 g/day) protein intakes [OR: 0.70 (95%CI: 0.48, 1.01)], but this estimate was driven by the significantly reduced risk observed in DNBC only [OR: 0.21 (95%CI: 0.07, 0.61)], not MoBa (OR: 0.92 (95%CI: 0.52, 1.65)), which points towards a chance finding.

Additional and more exploratory analyses also showed that the association for preterm delivery was driven by late (≥34 to <37 weeks), but not early (≥32 to <34 weeks) or very early (<32 weeks) preterm, delivery. The effect estimates on preterm delivery were not sensitive to the use of ultrasound vs. date of last menstrual period. We also examined the associations between intake of dairy protein (milk, yoghurt, cheese and butter) and preterm delivery. Overall, these were similar to the results for total proteins, but the effect estimate for preterm delivery was slightly stronger [OR: 1.17 (95%CI: 1.05, 1.30)] when examining very high intake of dairy proteins (>50 g, ~7% having intake at that level) vs. more moderate intake (20–30 g/day, 31% having intake at that level). 

## 4. Discussion

Contrary to previous findings from a randomized dietary intervention from the 1970s on high-protein supplementation in pregnancy [1], which has since influenced reviews on this subject and dietary recommendations [2,3,4,5,6,7], we found no indications of increased risk of growth restrictions or fetal and neonatal deaths at high protein intakes. High protein intake was modestly associated with risk of preterm delivery.

Preterm deliveries and low birth weight are risk factors for both perinatal mortality and morbidity as well as later physical and mental disabilities [23,24,25]. While protein is crucial for optimal intrauterine growth and development, the optimal range is less known. In the Harlem Trial [1], the supplement consisted of 40 g of protein in the form of casein. This amount is equivalent to ~1.4 L of cow’s milk, corresponding to around half the recommended protein intake for the pregnant women in our study population [4]. The supplementation in the Harlem Trial came out on top of the habitual protein intake, corresponding to ~100 g in total. The supplementation also included a number of vitamins and minerals, which were, with the exception of retinol (6000 retinol equivalents), well below the upper intake (UI) limits [4]. The supplementation may have been unbeneficial for all of the Harlem Trial outcomes and could relate to more than high protein intake only, such as specific effects of the protein type, casein, or combined effects of high protein and, e.g., retinol. Although speculative, a modest adverse effect of the supplementation may have, in the 1970s, also produced worse outcomes, in terms of offspring survival, than would occur in today’s setting with routine antenatal and ultrasound scanning that captures fetuses at risk, providing further monitoring and intervention. In light of our results, the role of a chance finding in the Harlem Trial also appears plausible.

The effect size of our finding for preterm birth was modest and its relevance should be subject to careful interpretation. It is, however, worth noting that our estimate for high intake [OR: 1.10 (1.01, 1.19)] was of similar magnitude as the effect estimate of preterm delivery in the Harlem Trial [relative risk of 1.14 (95%CI: 0.83; 1.56)], as synthesized from the original data [2]. Higher risk of preterm delivery has also been reported in a recent prospective Chinese study among subjects with relatively high intake of protein from dairy [26]. Regarding the possible mechanism by which protein intake might affect the length of gestation, previous studies have found that protein, especially from animal sources, is related to higher levels of inflammatory markers in serum during pregnancy [27,28], a condition that has been related to higher risk of preterm delivery [29,30].

The lower mean birth weight and increased risk of having low-birth weight infants observed among women with protein intake below 60 g/day in our study are in line with previous interventions showing beneficial effects of balanced protein supplementation in undernourished populations [2]. Standardized for weight, the women in the <60 g/day protein group in our study had a median intake of 0.87 g/kg body weight per day. The estimated average requirement for pregnant women according to the Institute of Medicine is 0.88 g/kg body weight per day, which is the lowest level of intake needed to account for losses of nitrogen from the body. There are some recent suggestions that the average protein requirements for pregnant women are somewhat higher [31], particularly in late gestation. In this context, it appears likely that some of the women in our low-protein group (<60 g/day) may have had insufficient intake to support optimal fetal growth.

Our study has several strengths. Firstly, the prospective design and large statistical power made it possible to examine associations with rare obstetric outcomes with some precision. The sample size of each cohort also allowed us to explore the stability of our findings separately in each country. This was important, as dietary and certain lifestyle habits (including smoking) are known to differ between the two countries and reproducing the estimate in two independent settings allows for more robust conclusions. Our outcome variables were extracted from national birth registries which use standard procedures for registration and where in-house quality is constantly monitored.

Concerning limitations, the fact that our study is observational means that we cannot rule out the influence of unadjusted confounder(s) and residual confounding. Another limitation is that the dietary assessment in the two cohorts did not cover the complete pregnancy period. However, studies with repeated dietary assessment have shown that dietary habits in pregnancy remain relatively stable [32,33]. With respect to the interpretation of our findings, a direct comparison between our study results and those reported in the Harlem Trial is hampered by important differences in the two study populations and study design. The women recruited into the Harlem Trial were recruited from a poor black African American community and those eligible for inclusion had to have at least one of the following risk factors for having a low-birth weight infant: (1) low pre-pregnancy weight (<110 pounds), (2) low weight gain at the time of recruitment, (3) at least one previous low-birth weight infant and (4) protein intake of <50 g as derived from one 24-h dietary recall. Relatively few women in our well-nourished Nordic population would have met one or more of these conditions. In addition, our study examined dietary, but not supplemental, protein, and these two ways of consumption have important differences. For example, high dietary protein intake from foods of animal origin is associated with higher intake of fats and certain minerals and vitamins including vitamin B6, B12, iron and zinc. High intake of proteins from plant sources is, however, usually associated with higher intake of carbohydrates and fibers and a different micronutrient profile. Our dietary intake estimates also reflect aggregated protein intake over the whole day. In contrast, protein supplementation usually means consumption of proteins largely in the absence of other macronutrients, and much larger amounts of proteins can be consumed as supplements in liquid form over a short period compared to normal dietary intake, which may have induced more metabolic strain [34]. In summary, given these important differences, the null findings from our Nordic study population do not reject the possibility that the effect of high-protein supplementation in the Harlem Trial had deleterious effects on fetal growth and neonatal mortality in that low-income, high-risk population [1].

## 5. Conclusions

In contrast to the results from the Harlem Trial, the results from this large prospective study, conducted in a well-nourished population, do not support a link between high dietary intake of proteins in pregnancy and growth retardation and fetal or neonatal deaths. However, in line with the results of the Harlem Trial, we found a modest, but significantly increased, risk of preterm delivery. Taken together, existing evidence suggests that for healthy pregnant women, high-protein diets have no clear benefits, and care should be taken before advocating extremely high intakes.

## Figures and Tables

**Table 1:** Characteristics of participants in the Danish National Birth Cohort (DNBC, 1996–2002) and the Norwegian Mother, Father and Child Cohort study (MoBa, 2002–2008).

	DNBC *n* = 60,141	MoBa *n* = 66,302	Combined *n* = 126,443
Maternal Characteristics			
Age, years	30.3 (4.2)	30.7 (4.6)	30.5 (4.4)
Pre-pregnancy BMI, kg/m^2^	23.5 (4.2)	24.1 (4.3)	23.8 (4.2)
<18.5	4.0	2.8	3.8
18.5–24.9	64.4	61.2	62.7
25–29.9	18.2	20.8	19.5
>30	7.2	8.9	8.1
Missing	6.1	6.3	6.3
Height (cm)	169 (6)	168 (6)	168 (6)
Education, %			
Less than basic studies	0.2	6.6	3.6
High school	24.0	24.0	24.1
College or higher education	50.4	63.4	57.1
Missing	25.4	5.8	15.2
Marital status, %			
Married	93.5	92.6	93.0
Unmarried	1.8	2.2	2.0
Missing	4.7	5.2	5.0
Smoking, %			
Non-smoker	74.1	87.5	85.4
Occasional smoker	12.6	2.7	6.0
Smokes daily	12.3	5.3	5.8
Missing	1.0	4.5	2.8
Parity, %			
0	55.2	46.7	50.7
1	31.1	34.7	33.0
2	11.3	15.0	13.3
3+	2.4	3.6	3.0
Energy intake (MJ/d)	10.0 (2.5)	9.7 (2.6)	9.9 (2.6)
Protein intake (% of Energy)	15.7 (2.4)	15.4 (2.1)	15.6 (2.3)
Protein intake (g/day)	92 (24)	87 (21)	89 (23)
Animal protein (g/day)	60 (19)	72 (20)	66 (21)
As dairy protein (g/day)	30 (15)	30 (17)	30 (16)
Plant protein (g/day)	32 (9)	15 (12)	23 (13)
Total fat intake (g/day)	84 (31)	80 (25)	82 (28)
Carbohydrates (g/day)	330 (87)	310 (93)	320 (91)
Obstetric outcomes			
Birth weight (g)	3587 (560)	3583 (555)	3591 (566)
Preterm delivery (<37 weeks), %	4.60	4.92	4.77
Low birth weight (<2500 g), %	2.85	3.01	2.93
Fetal death, %	0.31	0.26	0.28
Neonatal death, %	0.19	0.16	0.17

**Table 2:** Associations between maternal protein intake and birth weight for all births and for preterm (<37 weeks) births only. Mean differences (Δ) in birth weight across categories of protein intake relative to the reference (80–90 g) category are shown. The Danish National Birth Cohort (DNBC, 1996–2002) and the Norwegian Mother, Father and Child Cohort Study (MoBa, 2002–2008).

	All Births (*N* = 125,573)		Preterm (<37 Weeks) Only (*N* = 5898)	
	Unadjusted	Adjusted ^1^	Unadjusted	Adjusted ^1^
Protein ^2^	Δ (in Grams) (95%CI)	Δ (in Grams) (95%CI)	Δ (in Grams) (95%CI)	Δ (in Grams) (95%CI)
<60 g	−127 (−157, −97)	−51 (−74, −28)	−234 (−391, −76)	−128 (−230, −27)
60–70 g	−44 (−59, −31)	−20 (−31, −10)	9 (−69, 89)	28 (−22, 79)
70–80 g	−16 (−25, −8)	−6 (−13, 0)	−4 (−54, 46)	9 (−23, 40)
80–90 g	Referent	Referent	Referent	Referent
90–100 g	9 (1, 17)	9 (3, 15)	43 (−5, 90)	35 (4, 66)
>100 g	7 (−2, 17)	18 (10, 25)	54 (−1, 111)	31 (−6, 67)
*p* for effect ^3^	<0.0001	<0.0001	0.005	0.01

Δ: mean difference in birth weight in grams per day compared to the reference. ^1^ Adjusted for cohort, maternal age, parity, maternal smoking, pre-pregnancy body mass index, height, education, marital status, total energy intake and gestational age. ^2^ Across categories from low (<60 g) to high (>100 g) protein intakes, the corresponding median (10th–90th percentiles) protein intake relative to body weight (in g/kg) was 0.9 (0.6–1.1), 1.0 (0.8–1.3), 1.2 (0.9–1.4), 1.3 (1.0–1.6), 1.4 (1.1–1.7) and 1.6 (1.2–2.0), respectively. ^3^ F test type III.

**Table 3:** Associations between maternal protein intake and risk of preterm delivery and low birth weight. The associations are presented in terms of odds ratios (ORs) using the 80–90 g category as the reference. The Danish National Birth Cohort (DNBC, 1996–2002) and the Norwegian Mother, Father and Child Cohort study (MoBa, 2002–2008).

		Unadjusted	Adjusted ^1^
Protein ^2^	Cases (%)/N	OR (95%CI)	OR (95%CI)
*N* = 126,144		Preterm delivery (<37 weeks)	
<60 g	81 (5.9%)/1378	1.31 (1.04, 1.64)	1.17 (0.93, 1.47)
60–70 g	365 (5.0%)/7280	1.10 (0.98, 1.24)	1.06 (0.94, 1.19)
70–80 g	1283 (4.9%)/26,034	1.08 (1.01, 1.16)	1.07 (1.00, 1.15)
80–90 g	1949 (4.6%)/42,674	1.00	1.00
90–100 g	1454 (4.7%)/31,257	1.02 (0.95, 109)	1.02 (0.95, 1.10)
>100 g	884 (5.1%)/17,521	1.11 (1.02, 1.21)	1.10 (1.01, 1.19)
*p* for effect ^3^	(χ^2^, type III)	0.02	0.18
*N* = 125,624		Low birth weight (<2.5 kg)	
<60 g	75 (5.5%)/1368	1.97 (1.55, 2.50)	1.59 (1.25 2.03)
60–70 g	264 (3.6%)/7253	1.28 (1.12, 1.47)	1.17 (1.02, 1.34)
70–80 g	778 (3.0%)/25,944	1.05 (0.96, 1.15)	1.02 (0.93, 1.12)
80–90 g	1219 (2.9%)/42,536	1.00	1.00
90–100 g	839 (2.7%)/31,092	0.94 (0.86, 1.03)	0.95 (0.87, 1.04)
>100 g	508 (2.9%)/17,431	1.02 (0.92, 1.13)	1.01 (0.90, 1.12)
*p* for effect ^3^	(χ^2^, type III)	<0.0001	0.0005

^1^ Adjusted for cohort, age, parity, maternal smoking, pre-pregnancy body mass index, height, education, marital status, infant sex and total energy intake. ^2^ Across categories from low (<60 g) to high (>100 g) protein intakes, the corresponding median (10th–90th percentiles) protein intake relative to body weight (in g/kg) was 0.9 (0.6–1.1), 1.0 (0.8–1.3), 1.2 (0.9–1.4), 1.3 (1.0–1.6), 1.4 (1.1–1.7) and 1.6 (1.2–2.0), respectively. ^3^ F test type III.

**Table 4:** Extreme protein intake in relation to birth weight among all infants and preterm infants only. Mean differences (Δ) in birth weight across categories of protein intake relative to the reference (80–90 g) category are shown. The Danish National Birth Cohort (DNBC, 1996–2002) and the Norwegian Mother, Father and Child Cohort study (MoBa, 2002–2008).

	All Births (*N* = 125,573) ^1^		Preterm (<37 Weeks) Only (*N* = 5898) ^1^	
Protein ^2^	No. Referent/No. High	Δ (in Grams) (95%CI)	No. Referent/No. High	Δ (in Grams) (95%CI)
80–90 g vs. >100 g	42,593 vs. 17,455	18 (10, 25)	1919/864	31 (−6, 67)
80–90 g vs. >105 g	42,593 vs. 9428	16 (6, 26)	1919/482	38 (−9, 82)
80–90 g vs. >110 g	42,593 vs. 4842	10 (−3, 22)	1919/224	44 (−16, 105)
80–90 g vs. >115 g	42,593 vs. 2319	22 (5, 41)	1919/114	94 (9, 180)
80–90 g vs. >120 g	42,593 vs. 1086	13 (−12, 39)	1919/59	99 (−17, 216)

Δ: mean difference in birth weight in grams per day compared to the reference. ^1^ Adjusted for cohort, maternal age, parity, maternal smoking, pre-pregnancy body mass index, height, education, marital status, total energy intake and gestational age. ^2^ Protein intake (median (10th–90th percentiles)) relative to body weight (in g/kg) was 1.3 (1.0–1.6) in the 80–90 g group; 1.6 (1.2–2.0) in the >100 g group; 1.7 (1.3–2.0) in the >105 g group; 1.7 (1.3–2.1) in the >110 g group; 1.8 (1.4–2.2) in the >115 g group; and 1.9 (1.4–2.3) in the >120 g group.

**Table 5:** Extreme protein intake in relation to preterm birth and low birth weight. The associations are presented in terms of odds ratios (ORs) using the 80–90 g category as the reference. The Danish National Birth Cohort (DNBC, 1996–2002) and the Norwegian Mother, Father and Child Cohort study (MoBa, 2002–2008).

	Preterm Delivery (<37 Weeks) ^1^		Low Birth Weight (<2.5 kg) ^1^	
Protein ^2^	N/Cases (%)	OR (95%CI)	N/Cases (%)	OR (95%CI)
80–90 g vs. >100 g	42,674/1949 (4.6%) vs. 17,521/884 (5.1%)	1.10 (1.01, 1.19)	42,536/1219 (2.9%) vs. 17,431/508 (2.9%)	1.01 (0.90, 1.12)
80–90 g vs. >105 g	42,674/1949 (4.6%) vs. 9474/499 (5.3%)	1.14 (1.03, 1.27)	42,536/1219 (2.9%) vs. 9417/286 (3.0%)	1.04 (0.91, 1.19)
80–90 g vs. >110 g	42,674/1949 (4.6%) vs. 4872/255 (5.2%)	1.13 (0.98, 1.29)	42,536/1219 (2.9%) vs. 4835/149 (3.1%)	1.05 (0.89, 1.25)
80–90 g vs. >115 g	42,674/1949 (4.6%) vs. 2342/120 (5.1%)	1.09 (0.89, 1.31)	42,536/1219 (2.9%) vs. 2317/71 (3.1%)	1.02 (0.80, 1.31)
80–90 g vs. >120 g	42,674/1949 (4.6%) vs. 1094/59 (5.4%)	1.13 (0.86, 1.47)	42,536/1219 (2.9%) vs. 1086/32 (3.0%)	0.95 (0.66, 1.35)

^1^ Adjusted for cohort, maternal age, parity, maternal smoking, pre-pregnancy body mass index, height, education, marital status, infant sex and total energy intake. ^2^ Protein intake (median (10th–90th percentiles)) relative to body weight (in g/kg) was 1.3 (1.0–1.6) in the 80–90 g group; 1.6 (1.2–2.0) in the >100 g group; 1.7 (1.3–2.0) in the >105 g group; 1.7 (1.3–2.1) in the >110 g group; 1.8 (1.4–2.2) in the >115 g group; and 1.9 (1.4–2.3) in the >120 g group.

**Table 6:** Associations between maternal protein intake and risk of fetal and neonatal deaths. The associations are presented in terms of odds ratios (ORs) using the 80–90 g category as the reference. The Danish National Birth Cohort (DNBC, 1996–2002) and the Norwegian Mother, Father and Child Cohort study (MoBa, 2002–2008).

		Unadjusted	Adjusted ^1^
Protein ^2^	Cases (%)/N	OR (95%CI)	OR (95%CI)
*N* = 126,443		Fetal death	
<60 g	3 (0.22%)/1381	0.73 (0.23, 2.30)	0.78 (0.24, 2.58)
60–70 g	16 (0.22%)/7301	0.74 (0.44, 1.24)	0.88 (0.51, 1.52)
70–80 g	79 (0.30%)/26,110	1.02 (0.77, 1.35)	1.18 (0.88, 1.58)
80–90 g	127 (0.30%)/42,782	1.00	1.00
90–100 g	95 (0.30%)/31,314	1.02 (0.78, 1.33)	1.02 (0.77, 1.35)
>100 g	40 (0.23%)/17,555	0.77 (0.54, 1.10)	0.70 (0.48, 1.01)
*p* for effect ^3^	(χ^2^, type III)	0.52	0.22
*N* = 126,081		Neonatal death	
<60 g	1 (0.01%)/1378	0.47 (0.07, 3.38)	0.41 (0.06, 2.99)
60–70 g	18 (0.27%)/7285	1.59 (0.95, 2.69)	1.54 (0.91, 2.61)
70–80 g	45 (0.17%)/26,031	1.12 (0.77, 1.63)	1.14 (0.78, 1.66)
80–90 g	66 (0.15%)/42,655	1.00	1.00
90–100 g	56 (0.18%)/31,217	1.16 (0.81, 1.66)	1.12 (0.78, 1.60)
>100 g	34 (0.19%)/17,515	1.26 (0.83, 1.90)	1.11 (0.73, 1.70)
*p* for effect ^3^	(χ^2^, type III)	0.40	0.61

^1^ Adjusted for cohort, age, parity, maternal smoking, pre-pregnancy body mass index, height, education, marital status, infant sex and total energy intake. ^2^ Across categories from low (<60 g) to high (>100 g) protein intakes, the corresponding median (10th–90th percentiles) protein intake relative to body weight (in g/kg) was 0.9 (0.6–1.1), 1.0 (0.8–1.3), 1.2 (0.9–1.4), 1.3 (1.0–1.6), 1.4 (1.1–1.7) and 1.6 (1.2–2.0), respectively. ^3^ F test type III.

## Data Availability

The consent given by the participants does not open for storage of data on an individual level in repositories or journals. Researchers who want access to data sets for replication should submit an application. Access to data sets requires approval from ethical committees and an agreement with the cohorts.

## Supplementary Materials

The following are available online at https://www.mdpi.com/2072-6643/13/2/440/s1, Figure S1: Flowchart for inclusion of participants in the combined study from the two cohorts: Danish National Birth Cohort (DNBC) and the Norwegian Mother, Father and Child Cohort (MoBa), Table S1: Maternal protein intake in relation to preterm delivery and low birth weight in the Danish National Birth Cohort (1996–2002) and the Norwegian Mother, Father and Child Cohort study (2002–2008). Table S2. Associations between maternal protein intake and risk of preterm delivery and low birth weight stratified by pre-pregnancy body mass index.
