# Supplementary material for: Old Question Revisited: Are High-Protein Diets Safe in Pregnancy?
Source: Nutrients. 2021 Jan 29;13(2):440. doi: 10.3390/nu13020440 (PMC7911198; doi:10.3390/nu13020440)
Supplement: Supplementary file 1 [file nutrients-13-00440-s001.pdf]

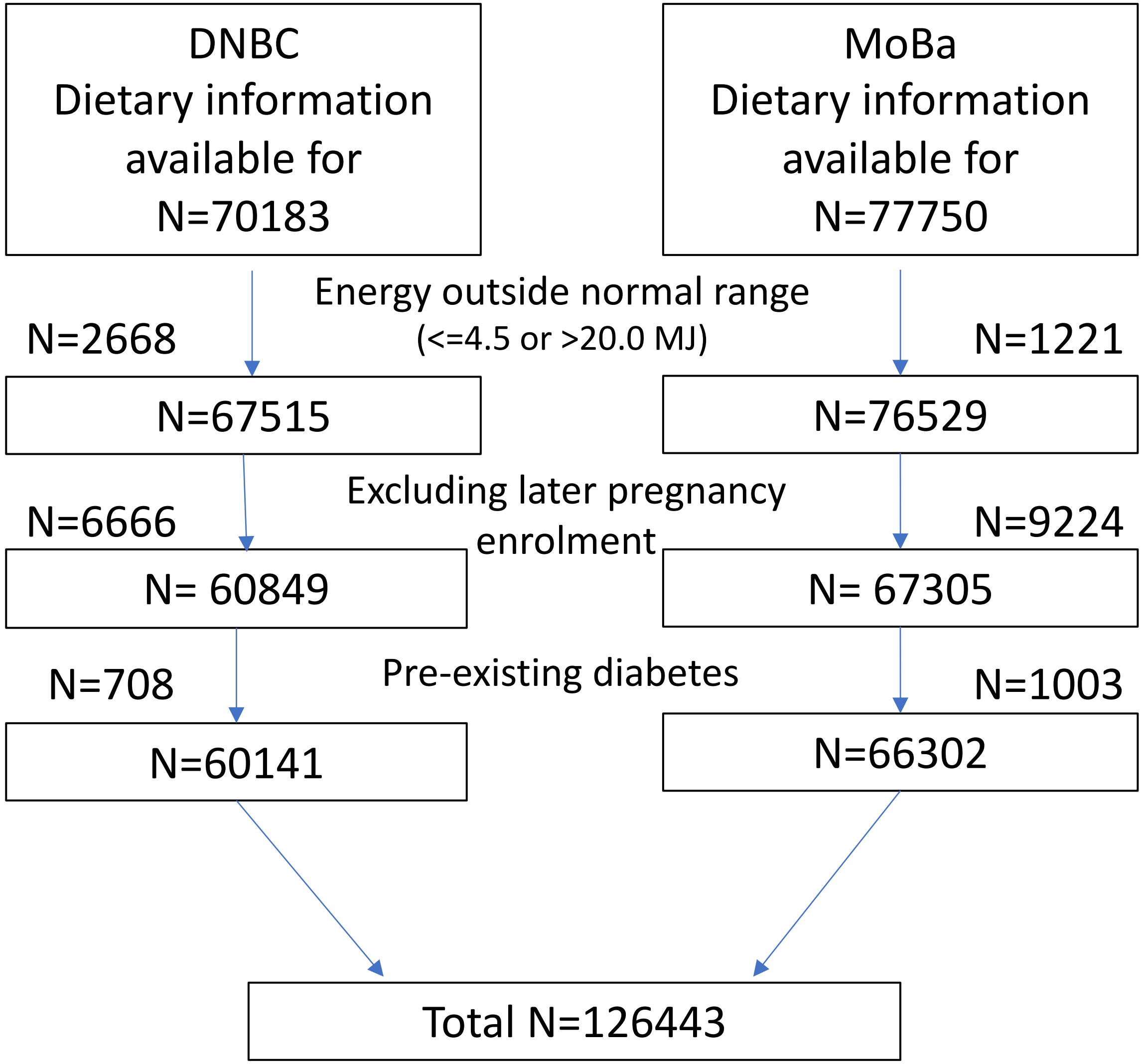

Supplemental Figure: Flowchart for inclusion of participants in the combined study from the two cohorts; Danish National Birth Cohort (DNBC) and the Norwegian Mother and Child Cohort (MoBa).
